# Return to work after medical rehabilitation in Germany: influence of individual factors and regional labour market based on administrative data

**DOI:** 10.1186/s12651-023-00330-1

**Published:** 2023-01-20

**Authors:** Christian Hetzel, Sarah Leinberger, Rainer Kaluscha, Angela Kranzmann, Nadine Schmidt, Anke Mitschele

**Affiliations:** 1 Institute for Quality Assurance in Prevention and Rehabilitation at the German Sport University in Cologne, Eupener Str. 70, 50933 Cologne, Germany; 2 Institute for Research in Rehabilitation Medicine at Ulm University, Bad Buchau, Germany; 3 Federal German Pension Insurance, Berlin, Germany

**Keywords:** Return to work, Labour market, Rehabilitation, Orthopaedic, Psychosomatic, I130, J210, J140

## Abstract

**Background:**

The influence of both individual factors and, in particular, the regional labour market on the return to work after medical rehabilitation is to be analyzed based on comprehensive administrative data from the German Pension Insurance and Employment Agencies.

**Method:**

For rehabilitation in 2016, pre- and post-rehabilitation employment was determined from German Pension Insurance data for 305,980 patients in 589 orthopaedic rehabilitation departments and 117,386 patients in 202 psychosomatic rehabilitation departments. Labour market data was linked to the district of residence and categorized into 257 labour market regions. RTW was operationalized as the number of employment days in the calendar year after medical rehabilitation. Predictors are individual data (socio-demographics, rehabilitation biography, employment biography) and contextual data (regional unemployment rate, rehabilitation department level: percentage of patients employed before). The estimation method used was fractional logit regression in a cross-classified multilevel model.

**Results:**

The effect of the regional unemployment rate on RTW is significant yet small. It is even smaller (orthopaedics) or not significant (psychosomatics) when individual employment biographies (i.e., pre-rehabilitation employment status) are inserted into the model as the most important predictors. The interaction with pre-rehabilitation employment status is not substantial.

**Conclusions:**

Database and methods are of high quality, however due to the nonexperimental design, omitted variables could lead to bias and limit causal interpretation. The influence of the labour market on RTW is small and proxied to a large extent by individual employment biographies. However, if no (valid) employment biographies are available, the labour market should be included in RTW analyses.

**Supplementary Information:**

The online version contains supplementary material available at 10.1186/s12651-023-00330-1.

## Introduction

### Rehabilitative interventions

For people with health restrictions or disabilities, participation in working life is an important individual and social goal. One means of achieving this is through rehabilitation. The WHO defines rehabilitation as “a set of interventions designed to optimize functioning and reduce disability in individuals with health conditions in interaction with their environment” (Word Health Organization 2020). In 2019, approximately 2.41 billion individuals worldwide had health conditions that would benefit from rehabilitation, which counters the common view of rehabilitation as a service for the few. This number increased by 63 percent from 1990 to 2019. The illness category constituting the largest share of this figure was musculoskeletal disorders (approximately 1.71 billion people), with lower back pain being the most prevalent condition in 134 of the 204 countries analyzed (Cieza et al. 2020). It is evident that many rehabilitative interventions are cost-effective (Howard-Wilsher et al. 2016; Shields et al. 2018; Krischak et al. 2019; Miyamoto et al. 2019), because chronification can be counteracted and the ability to work can be maintained.

In view of the demographic change and prolonged working lives, the proportion of older employees is increasing in most economies, and thus also the number of employees with poor health and functional limitations (van den Berg et al. 2010). Therefore, one major public health goal should be avoiding premature termination of work due to poor health using primary prevention, rehabilitation and RTW strategies. These factors will gain relevance in working life, as for example the aim of medical rehabilitation is a continuous participation in working life. Understanding which factors favour or slow down rehabilitation outcomes can help to develop appropriate political concepts to support people return to work (RTW) after a health shock (Young et al. 2005). From a social point of view, social security contribution payments are maintained.

### Institutional background in Germany

Implemented rehabilitation programmes vary significantly from country to country (International Social Security Association 2001; Belin et al. 2016). In contrast to outpatient interventions in other countries, in Germany interventions are mostly conducted as 3-week or 4-week inpatient programmes in specialized rehabilitation departments, but increasingly as an outpatient or semi-inpatient, close to the patient’s home. Rehabilitation is a part of the social insurance system, mostly provided by the German statutory pension insurance (GPI), German statutory health insurance or private health insurance. The German statutory health insurances provide outpatient or inpatient medical rehabilitation (§ 40 SGB V) if treatment interventions alone are not adequate to cope with the consequences of illness. To counteract the effects of a psychological or physical disorder on earning capacity, the GPI provides benefits for medical and vocational rehabilitation (§ 9, § 10 SGB VI). For example, if certain formal prerequisites are met, a patient is entitled to apply for medical rehabilitation at the GPI. If this application is confirmed by the GPI, the patient has the legal right to take part in a medical rehabilitation programme. The fundamental principle applies: rehabilitation has priority over pension. Before someone can receive a pension owning to reduced earning capacity, the GPI will check if rehabilitation can be carried out. The GPI is the main provider of medical rehabilitation in Germany with over 1 million medical rehabilitation programmes in 2018 and approximately 5 billion euros spent in this sector (Deutsche Rentenversicherung Bund 2019).

Vocational rehabilitation programmes can be provided on their own or as a supplement to a completed medical rehabilitation programme. There are programmes designed to keep individuals in their job, but there are also education and training interventions designed to offer entirely new career prospects.

How much is income replacement during medical rehabilitation? In principle, employees are entitled to a continued payment of their wage or salary for up to six weeks due to illness. If there is no longer any entitlement to this, the GPI pays transitional benefits (68 percent of the net wage) for that time. Individuals are obliged to pay compulsory contributions into the GPI, in particular if they receive a wage or salary including short-term sickness. People in marginal employment, the long-term unemployed and old-age-pensioners are exempt from paying compulsory contributions. However, voluntary contributions can be paid. The number of employment days referred to in this article is based on an individual receiving a wage or salary above the marginal employment level including short-term sickness and therefore these individuals pay compulsory contributions into the GPI.

### Return to work after medical rehabilitation

One of the main goals of rehabilitative interventions financed by the GPI is the partial or complete (re-)integration into working life (Deutsche Rentenversicherung Bund 2019). However, the patients’ RTW depends not only on the quality of the rehabilitation programme itself but also on personal and contextual factors. There has been surprisingly little research into this area and conceptual and empirical data to substantiate this finding is rather inconsistent. This is especially true for the influence of the regional labour market on RTW after medical rehabilitation—in the indication area of “depression”, there has been an explicit demand for further research to be conducted (Ervasti et al. 2017).

The regional labour market is only partially taken into consideration as an influencing factor in RTW concepts, e.g., as an environmental contextual factor of the International Classification of Functioning, Disability and Health, as a facet of the theory of perceived insecurity (Stewart et al. 2012) or as the macro level in system-theoretical models (Loisel et al. 2005). The matching theory of supply and demand on labour markets (Petrongolo and Pissaridēs 2001) also implicitly postulates a connection between the regional labour market and employment. The theory describes that employers’ wage offers partially depend on the wage-lowering effect of high regional unemployment (Müller and Blien 2001).

Empirical studies take the regional labour market into account to some extent. Studies from unemployment contexts (Hirschenauer 2013), vocational rehabilitation (Hetzel and Streibelt 2016; Reims and Tophoven 2018; Echarti et al. 2020) and medical rehabilitation (Kaluscha et al. 2013) indicate that the regional labour market decreases RTW. In these contexts, the labour market is operationalized either indirectly via assignment to a regional unit (Leinonen et al. 2019; Echarti et al. 2020) or directly via characteristics of the regional labour market, such as the unemployment rate (Kaluscha et al. 2013; Hetzel 2015; Hetzel and Streibelt 2016; Reims and Tophoven 2018). In our opinion, there is evidence for vocational rehabilitation (Hetzel and Streibelt 2016; Reims and Tophoven 2018; Echarti et al. 2020), but not for medical rehabilitation. To the best of our knowledge, only Kaluscha et al. (2013) use labour market conditions predicting RTW in medical rehabilitation. Based on randomly selected administrative data of the GPI between 2002 and 2009, they explore labour market effects using federal states in Germany as regional units and unemployment rates with different standardizations. They conducted an extensive data-driven selection of 12 predictors. The result was that the labour market improved model fit in some models, but a clear operationalization and estimation of the size of an effect was not possible. Other studies neglect to consider the regional labour market (International Social Security Association 2001; Celsing et al. 2012; Howard-Wilsher et al. 2016; Ervasti et al. 2017; Odgaard et al. 2018; Nevala et al. 2019). Either there is no labour market effect or an existing effect is not modeled in an appropriate way. This could possibly be explained by the fact that intervention studies are often realized within a small geographical range, which means that there is little variance in the regional labour market and thus its influence might remain undetected. In addition, the labour market effect could be masked by systemic conditions such as unemployment benefits or early retirement (International Social Security Association 2001; Belin et al. 2016). Individual characteristics could also obscure existing effects if they are correlated to the labour market (e.g., recent periods of unemployment (Celsing et al. 2012)). If the focus is on returning to a job that existed before rehabilitation, job-related rather than labour market-related characteristics are likely to be significant predictors (International Social Security Association 2001; Jansen et al. 2021).

From a research perspective, controlling the effects of confounding variables is important to determine intervention effects with minimal bias and to enable fair benchmarking opportunities between regions or providers, for example in the context of GPI quality management (Zeisberger et al. 2019). To achieve fair benchmarking opportunities, the effect of characteristics for which the rehabilitation department is not responsible (e.g., the labour market at the patient’s place of residence) are to be considered in the assessment of treatment outcome (risk adjustment). If RTW is substantially influenced by the labour market, omitting this characteristic would lead to bias. Another reason to include the labour market is the limited availability of confounding variables in secondary data. Modeling labour market effects on RTW could substitute unobserved characteristics, such as employment biography or job conditions (Bülau et al. 2016), and thus increase the model quality.

All in all, unlike vocational rehabilitation, it is unclear whether there are labour market effects on the RTW in the context of medical rehabilitation. Evidence in this field can help to determine intervention effects with minimal bias and to enable fair benchmarking opportunities.

### Objective and hypotheses

**Fig. 1:**
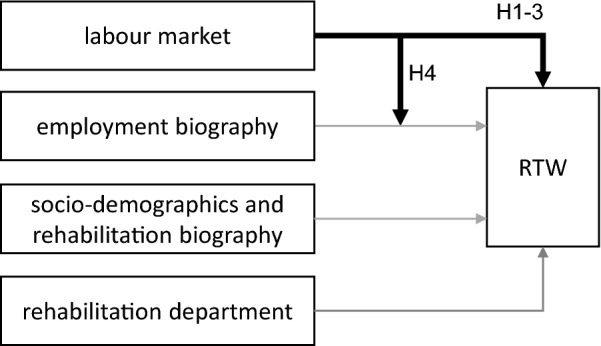
Effect relationships and hypotheses (H)

The objective of the present paper is to determine the influence of the regional unemployment rate on RTW after medical rehabilitation for the two largest indicators—orthopaedics and psychosomatics—using full administrative data from the GPI based on a large number of observations. The following hypotheses will be tested (Fig. 1).

- *H1. A higher regional unemployment rate lowers RTW.*
  This expected negative effect is based on the concepts and empirical findings described above. In consistency with matching theory, it has to be examined whether an exponential correlation exists (Petrongolo and Pissaridēs 2001).
- *H2. The main effect of the regional unemployment rate on RTW is independent of socio-demographics and rehabilitation biography.*
  The main effect describes the extent to which the regional labour market influences the outcome variable directly, without the influences of other variables. For example, older persons might have more RTW restrictions than younger persons (Steiner 2017), but it is independent of labour market conditions. Therefore, the main effect of the regional unemployment rate should be similar to H1, when socio-demographics and rehabilitation biographies are accounted for*.*
- *H3. The main effect of the regional unemployment rate on RTW decreases when the employment biography is controlled for.*
  The independency described in H2 should be different for the characteristics of the individual employment biography. This is because they depend on past labour market conditions, which are also reflected in the current labour market. Therefore, employment biography should already include part of the model’s labour market effect, resulting in a reduced main effect of the unemployment rate compared to the models in H1 and H2.
- *H4. The labour market effect on RTW is more evident for patients who were unemployed before the rehabilitative intervention than for previously employed patients (interaction effect).*
  An interaction effect (Hayes 2013) describes that the influence of a predictor on the outcome variable depends on other confounding variables. After vocational rehabilitation, the regional labour market was found to moderate the influence of the prerehabilitation employment status on RTW (Hetzel and Streibelt 2016). There was a clearer dependency of RTW on labour market conditions among prerehabilitation unemployed patients than among prerehabilitation employed patients.

## Data, variables, and method

### Data

We use the rehabilitation statistics database (RSD) of the GPI (Deutsche Rentenversicherung 2015). The RSD contains administratively produced data from all the GPI institutions, in this case for medical rehabilitation programmes for the two largest indicators, orthopaedics and psychosomatics, in 2016. The database also contains all wages or salaries with social insurance contributions until 2017. The individual rehabilitation programme is linked to a certain rehabilitation department. We linked the regional unemployment rate for the patients’ labour market region (Bundesinstitut für Bau-, Stadt- und Raumforschung 2020) to their place of residence. These regional units (n = 257 labour market regions in Germany) are homogenized based on commuter interdependences (31.12.2015), i.e., there is a lot of commuting within a region and less commuting between regions. For the number of cases and exclusion criteria, see Table 1. Persons with missing data are 0.4 percent in psychosomatics and 0.5 percent in orthopaedics. We made a complete case analysis and did not impute data, especially as some variables, e.g., place of residence, cannot be reliably estimated.

**Table 1:** Number of cases and exclusion criteria

	Orthopaedics		Psychosomatics	
Patients who completed medical rehabilitation in 2016 (the last rehabilitation if more than one)	360,285		131,404	
− Exclusion based on theoretical considerations	52,653	14.6%	13,458	10.2%
Duration of rehabilitation: shorter than 7 days				
Special types of rehabilitation: contract performance^a^, aftercare, prevention, cancer, detoxification				
At the time of application: old-age pensioner, housewife/husband, not in working age (between 18 and 65 years), residence abroad/unknown				
In the year prior to application: no compulsory contributions to the GPI^b^				
In the observation period: death				
− Exclusion because of missing data	1652	0.5%	560	0.4%
= Final database	305,980	84.9%	117,386	89.3%

^a^For example cancer rehabilitation for the statutory health insurance, ^b^for example eligible long-term unemployed patients

### Variables

The analyzed outcome is the patients’ RTW, operationalized by the number of employment days in the first calendar year after medical rehabilitation.

We use the following predictors:

- individual characteristics and rehabilitation biography of the patient: (1) gender, (2) age (categories), (3) marital status, (4) migration, (5) vocational education, (6) place of residence in the area of former West Germany or the newly-formed German states (7), current intervention is post-hospital curative treatment (AHB)^1^, (8) current intervention is a special medical programme^2^, (9) additional payment claim after an individual income check (§ 32 SGB VI), (10) application for or receipt of pension for reduced earning capacity in temporal relation to the rehabilitation programme, and (11) number of rehabilitation programmes in the 4 years prior to the intervention.
- individual factors governing employment biography: (1) prerehabilitation employment status (employed/not employed in the 3rd month before rehabilitation start), (2) employment days in the first calendar year and (3) in the second calendar year prior to the rehabilitation programme (both in categories).
- labour market: regional unemployment rate at place of residence (in percent, not centred).
- rehabilitation department: percentage of patients with prerehabilitation employment status "employed" (in percent).

### Method

The data structure leads to dependencies, as patients are simultaneously categorized in both departments and in labour market regions. Therefore, we use a cross-classified multilevel model (Hox et al. 2018). To estimate the main effects (H1-3) and the interaction effect (H4), the models (M) are configured in blocks as follows:

- M1: cross-classified multilevel model with regional unemployment rate only
- M2: + patients’ personal characteristics and rehabilitation biography
- M3: + patients’ employment biography
- M4: + interaction "employment status 3 months before" with "regional unemployment rate”

In M1, we additionally test nonlinear associations. Therefore, we use squaring and logarithmising, respectively. The squaring operationalizes a u-shaped relationship between the unemployment rate and the outcome variable, and the logarithmising an inverse exponential relationship.

**Fig. 2:**
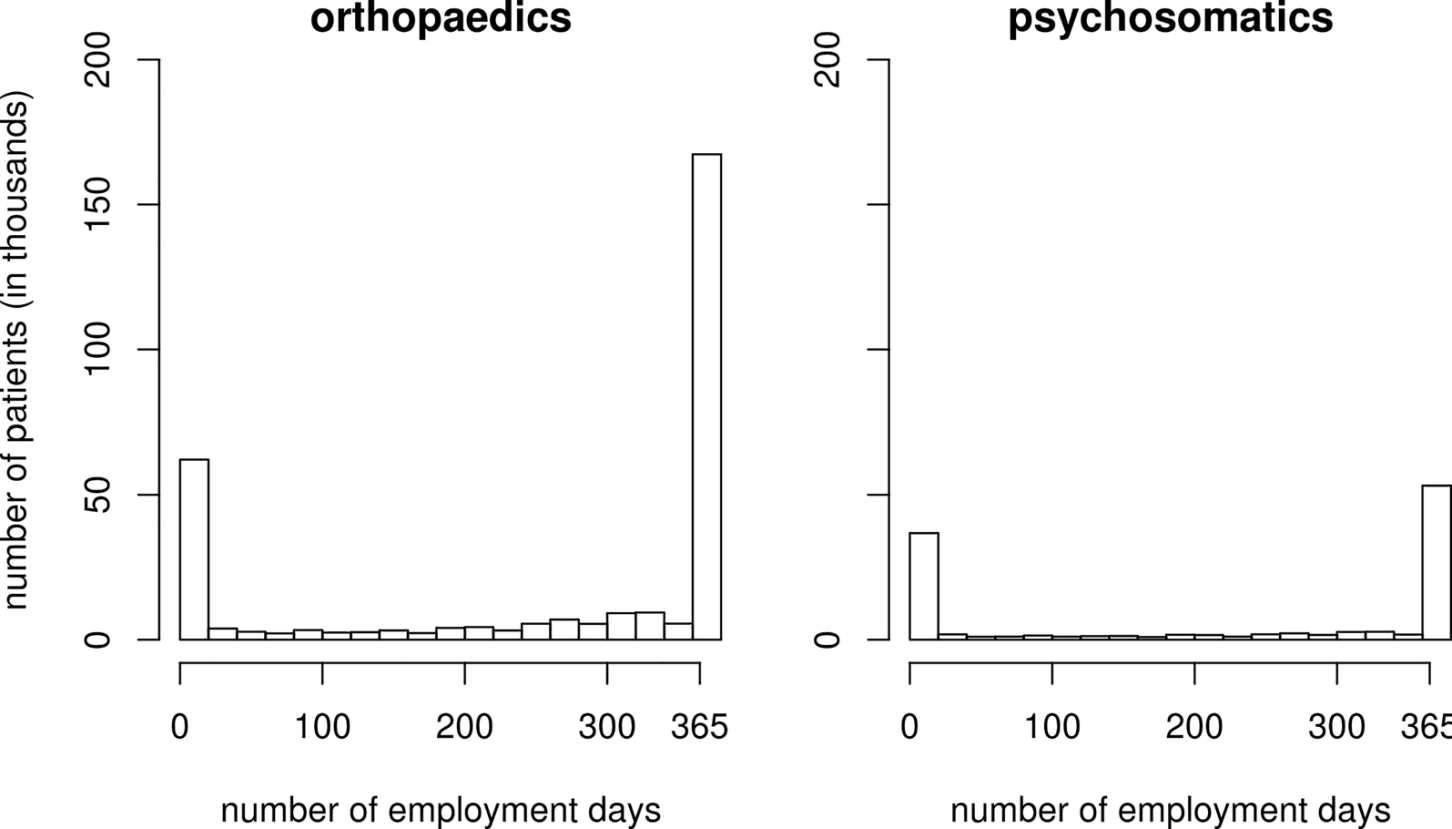
Number of employment days in the first calendar year after medical rehabilitation. See text for details

The outcome variable has a bimodal distribution in the interval between 0 and 365 days (see Fig. 2). Linear regressions would thus lead to distortions and a classification or dichotomization of the outcome variable would lead to loss of information. Therefore, we use fractional logit regression (FLR) as an estimation method. FLR models assume that the outcome variable is a proportion of the interval from 0 to 1, so we made a linear transformation of the outcome (dividing by 365 days). FLR was first described by Wedderburn (1974), generalized by McCullagh (1983) and rediscovered by Papke and Wooldridge (1996). FLR belongs to the family of generalized linear models, is based on quasi-maximum likelihood estimators, and is very similar to binary logistic regression. The basis of FLR is a binomial distribution, but with an additional parameter to estimate the deviatoric error variance in the data. This makes the method very flexible and does not require any special distributional assumptions. At the core is the logit link function, as in a binary logistic regression.

We report logits (b-coefficients) where negative logits describe a negative association. To quantify the effect size of the labour market, we use average marginal effects (AME) to describe the average effect of the labour market as the mean of the marginal effects across all observations. AME are reported in the unit of the outcome variable (days).^3^ For model fit, we use AIC and Pseudo-R^2^ (the squared correlation of expected and observed values).

## Results

### Descriptive results

The database contains 257 labour market regions, 589 rehabilitation departments and 305,980 individuals for orthopaedics, and 202 rehabilitation departments and 117,386 individuals for psychosomatics. For a description of the data, see Table 2. The RTW shows a bimodal distribution (Fig. 2). This means that the majority of patients either work 0 days or 365 days in the first calendar year after medical rehabilitation. This applies to both indication areas.

**Table 2:** Description of patients in orthopaedics and psychosomatics in 2016

	Orthopaedics (N = 305,980)	Psychosomatics (N = 117,386)
Gender		
Not female	50.7%	36.7%
Female	49.3%	63.3%
Age, in years		
< 25	0.9%	1.1%
25–30	2.3%	2.9%
31–35	3.4%	4.7%
36–40	5.0%	6.8%
41–45	7.6%	9.6%
46–50	14.8%	17.5%
51–55	23.0%	23.7%
56–60	26.0%	22.7%
61–65	17.0%	11.1%
Marital status		
Married	67.7%	58.8%
Single	15.4%	19.1%
Divorced	13.0%	16.3%
Widowed	2.6%	3.5%
n.a	1.4%	2.4%
Migration (country of birth* nationality)		
Germany*German	87.8%	88.9%
Other*other	3.4%	3.2%
Other*German	6.2%	5.5%
Germany*other	2.5%	2.5%
Vocational education		
No	37.8%	33.2%
Yes	62.2%	66.8%
Region (German states)		
Former West Germany	81.6%	85.1%
New states in Germany	18.4%	14.9%
Post-hospital curative treatment		
No	64.7%	100.0%
Yes	35.3%	0.0%
Special medical programmes		
Normal	87.0%	75.8%
Work-related	8.5%	23.6%
Behavioral	3.6%	0.0%
Other	0.9%	0.6%
Additional payment claim		
No	34.9%	15.5%
Yes	65.1%	84.5%
Application for reduced earning capacity pension		
No	97.6%	91.1%
Yes	2.4%	8.9%
Number of prior rehabilitations in the last 4 years		
0	76.7%	81.2%
1	13.4%	12.8%
2	7.8%	4.9%
3	2.1%	1.0%
Prerehabilitation employment status (3rd month before)		
Employed	77.0%	57.0%
Not employed	23.0%	43.0%
Employment days one calendar year before		
< 50	8.4%	13.8%
50–99	1.6%	3.2%
100–149	1.7%	3.4%
150–199	2.6%	4.6%
200–249	3.5%	5.4%
250–299	4.9%	6.0%
300–349	8.1%	9.0%
≥ 350	69.1%	54.7%
Employment days two calendar years before		
< 50	7.7%	7.9%
50–99	1.2%	1.8%
100–149	1.3%	1.9%
150–199	2.0%	2.8%
200–249	2.5%	3.2%
250–299	3.6%	4.0%
300–349	4.9%	5.6%
≥ 350	76.9%	72.7%
Prerehabilitation employment status (employed) on department level (%)		
Mean ± sd	67.73 ± 7.07	52.48 ± 8.81
Regional unemployment rate (%)		
Mean ± sd	5.93 ± 2.44	6.32 ± 2.38
Employment days one calendar year after		
Mean ± sd	256.51 ± 149.58	214.80 ± 164.45

### Orthopaedic rehabilitation

The models for orthopaedics are described in Table 3.

**Table 3:** Fractional logit regression models for RTW in orthopaedics

Predictors	M1		M2 (M1 + personal and rehabilitation biography)		M3 (M2 + employment biography)		M4 (M3 + interaction)	
	b {AME}	s.e	b {AME}	s.e	b {AME}	s.e	b {AME}	s.e
Unemployment rate (UR)	− 0.037 ***	0.004	− 0.040 ***	0.005	− 0.010 **	0.004	− 0.013 ***	0.004
	{ − 3.1}		{ − 2,8}		{ − 0.2}			
Prerehabilitation employment status [not employed, reference: employed]					− 1.272 *** { − 201.2}	0.012	− 1.338 *** { − 201.3}	0.029
UR * [not employed]							0.011 *	0.004
{AME, subgroup “employed”}							{ − 0.4}	
{AME, subgroup “not employed”}							{ 0.2}	
Random effects								
τ00, _labour market region_	0.01		0.01		< 0.01		< 0.01	
τ00, _rehabilitation departments_	0.05		0.05		0.01		0.01	
Pseudo-R^2^	0.019		0.144		0.363		0.363	
AIC	369,386		339,216		282,902		282,898	

Method is cross-classified fractional logit regression with n _labour market regions_ = 257, n _rehabilitation departments_ = 589, n _patients_ = 305,980, * p < 0.05, ** p < 0.01, *** p < 0.001; estimators for intercept, for personal/rehabilitation biography predictors and employment biography predictors in Additional file 1

*M* model, *b*  coefficients, *s.e.* standard error, *AME*  average marginal effects (in days), *τ*_*00*_  variance component of labour market region or rehabilitation department, *R*^*2*^  square of the correlation between the model’s predicted values and the actual values, *AIC*  Akaike-criterion

Without control variables (M1), the main effect of the unemployment rate is significant and in the expected direction, but weak: a 1 percent point higher unemployment rate reduces employment days by 3.1 days in the first calendar year after rehabilitation—averaged over all observations in the present sample (H1). Testing for nonlinear relationship of the regional unemployment rate (UR) with the outcome variable shows that there is no u-shaped relationship (UR: b = − 0.042, s.e. = 0.016, p = 0.009; UR^2^: b = 0.001, s.e. = 0.001, p = 0.715). Moreover, the relationship is linear rather than exponential because the log UR is significant (log UR: b = − 0.215 s.e. = 0.021, p < 0.001), but the model fit is slightly worse (AIC = 369,391) than for M1.

As expected, by adding the socio-demographics and rehabilitation biography (M2), the effect sizes remain very similar (H2).

By adding the employment biography (M3), the main effect of the regional unemployment rate still is significant, but weaker (AME only − 0.2 days) (H3).

**Fig. 3:**
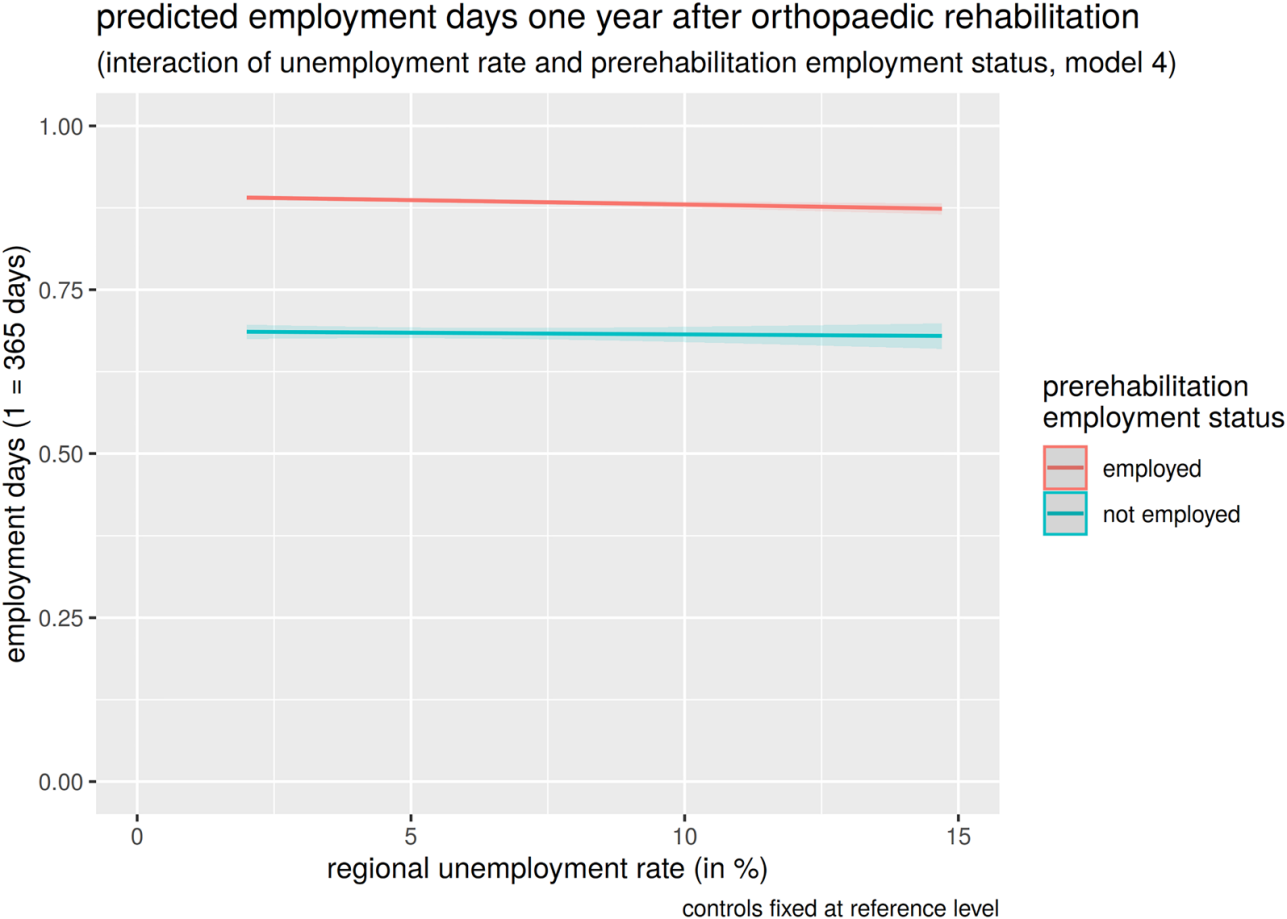
Interaction of unemployment rate and prerehabilitation employment status in orthopaedics

H4: The interaction of the unemployment rate with prerehabilitation employment status is ambiguous (M4). Arguments for including the interaction in the model are the significant interaction effect and the more favourable AIC in M4. Arguments against inclusion are that the AIC is only marginally lower than in M3, that R^2^ is the same in M3 and M4 and that the effect size of the interaction effect is low. The latter can particularly be seen in Fig. 3. Adding the interaction hardly changes the curve for the two groups, as they are still almost parallel. Apparently, the unemployment rate and the employment days are independent. The corresponding coefficients of M4 are shown in Table 3: for patients employed prerehabilitation, regional unemployment rate has a minimal effect (b = − 0.013, p = 0.001, AME = − 0.4 days) and, on the contrary, the effect for unemployed patients is significant (b = 0.011, p = 0.011). It should be emphasized that the interaction effect is very small.

The patients’ employment biography dominates the model, as seen from the clear shift of model R^2^ from model 2 to 3. The random effects are close to zero, indicating that both rehabilitation departments and the labour market are well operationalized. This means that the variance of the outcome variable which is due to the grouping of individuals into departments or labour market is almost completely modelled.

### Psychosomatic rehabilitation

The findings for psychosomatic rehabilitation are very similar to those for orthopaedics. In contrast, the findings for H3 are somewhat clearer, because the labour market effect is no longer significant when controlling for employment history. In addition, although the interaction effect (H4) remains very small, the statistics produce opposing effects for prerehabilitation unemployed (AME = 0.6 days) and employed individuals (AME = − 0.5 days). The models are shown in Table 4 — the graph for the interaction effect looks similar to Fig. 3 and is not shown here. The findings on linearity (H1) are also similar: no u-shaped relationship (UR: b = 0.019, s.e. = 0.021, p = 0.374; UR^2^: b = − 0.003, s.e. = 0.002, p = 0.070) and a linear rather than exponential relationship (log UR: b = − 0.100, s.e. = 0.027, p < 0.001, AIC = 157,166).

**Table 4:** Fractional logit regression models for RTW in psychosomatics

Predictors	M1		M2 (M1 + personal and rehabilitation biography)		M3 (M2 + employment biography)		M4 (M3 + interaction)	
	b {AME}	s.e	b {AME}	s.e	b {AME}	s.e	b {AME}	s.e
Unemployment rate (UR)	− 0.019 ^***^	0.004	− 0.025 ^***^	0.005	− 0.002	0.004	− 0.011 ^*^	0.005
	{ − 2.2}		{ − 2.2}		{ 0.0}			
Prerehabilitation employment status [not employed, reference: employed]					− 1.229 ^***^ { 209.9}	0.018	− 1.341 ^***^ { 209.9}	0.042
UR * [not employed]							0.018 ^**^	0.006
{AME, subgroup „employed “}							{ − 0.5}	
{AME, subgroup „not employed “}							{ 0.6}	
Random effects								
τ_00, labour market region_	0.01		0.01		< 0.01		< 0.01	
τ_00, rehabilitation departments_	0.13		0.12		0.04		0.04	
Pseudo-R^2^	0.029		0.206		0.386		0.386	
AIC	157,162		138,530		117,661		117,655	

Method is cross-classified fractional logit regression with n _labour market region_ = 257, n _rehabilitation departments_ = 202, n _patients_ = 117,386, * p < 0.05, ** p < 0.01, *** p < 0.001; estimators for intercept, for personal / rehabilitation biography predictors and employment biography predictors in Additional file 1

*M*  model, *b*  coefficients, *s.e.*  standard error, *AME*  average marginal effects (in days), *τ*_*00*_  variance component of labour market region or rehabilitation department, *R*^*2*^  square of the correlation between the model’s predicted values and the actual values, *AIC*  Akaike-criterion

### Robustness tests

We did some additional analyses to monitor the robustness of the labour market findings. We used different operationalizations of the outcome variable: (1) a dummy coded RTW with a cut off at 183 days which is the usual duration of the statutory probationary period of employment,^4^ and (2) RTW in days in the second calendar year after intervention. Furthermore, we also monitored findings withno change of the outcome variable: (3) subgroup analyses based on individual diagnosis (the three largest groups of main diagnosis at discharge) and (4) further predictors of the employment biography, which have proven to be the main predictor of RTW. The results in Table 5 show that the labour market AMEs are robust.

**Table 5:** Robustness tests

Outcome in calendar year	Method	Sample	Orthopaedics			Psychosomatics		
			AME for unemployment rate			AME for unemployment rate		
			M1	M2	M3	M1	M2	M3
RTW no/yes 1st	Linear regression^a^	All^b^	− 2.8	− 2.7	0.1	− 1.6	− 1.9	− 0.2
RTW days 2nd	FLR	All^b^	− 2.7	− 2.4	− 0.4	− 1.9	− 1.8	0.0
RTW days 1st	FLR	Subgroup back pain (n = 76,289)	− 3.9	− 3.4	− 0.5	na	na	na
		Subgroup endoprosthesis (n = 46,691)	− 3.5	− 1.6	− 0.2	na	na	na
		Subgroup depression (n = 66,945)	na	na	na	− 2.0	− 2.2	− 0.1
RTW days 1st	FLR	Added predictors M3: employment status in the 6th, 12th and 24th month before	na	na	− 0.2	na	na	0.0

Models (M) 1, 2 and 3 adjusted for the same predictors as before; *RTW*  return to work, *AME*  average marginal effects (in days); ^a^dichotomized with cut off 183 days; ^b^orthopaedics n = 305,980, psychosomatics n = 117,386

## Discussion

We determined the influence of the regional unemployment rate on RTW after medical rehabilitation for the two largest indication groups orthopaedics and psychosomatics. To the best of our knowledge, this was the first time that this was done explicitly using a representative database, in this case administrative data from the GPI for medical rehabilitation programmes in 2016. The core findings reveal that the regional unemployment rate has minimal effect on RTW. The effect is even smaller and near zero (orthopaedics) or not significant (psychosomatics) when individual employment biographies are added to the model as the most important predictors. The influence of the regional unemployment rate on RTW depending on the prerehabilitation employment status is not substantial.

The influence of the labour market is small, but still significant depending on the inclusion of further covariates. These findings are in line with the only other study on this topic known to us (Kaluscha et al. 2013), even though it was based on a different operationalization. The low effect size also explains the finding initially reported that most studies on RTW after medical rehabilitation do not even take the labour market into account. In view of the statistical significance of the labour market, an omission could nevertheless lead to (presumably small) biases.

The influence of the labour market seems to be smaller compared to vocational rehabilitation. This is suggested by findings on vocational education interventions that were also based on the RSD using similar regression methods but from a different year and with a different RTW operationalization (Hetzel and Streibelt 2016). The RTW range between the regions with the lowest and highest unemployment rates is about 30 percentage points and 7 percentage points respectively.^5^ No direct relation can be made to the study by Reims and Tophoven (2018). They analyzed vocational rehabilitation between 2009 and 2012 on behalf of the Federal Employment Agency (FEA) and report hazard ratios derived from event history analysis. However, as they used types of labour market regions, our findings are not comparable in this respect. The different labour market dependence on RTW can be explained by the fact that in vocational rehabilitation, the focus is usually on finding and starting a new job, while in medical rehabilitation, the focus is usually on returning to the old job. It could also be that the types of treatment used for medical rehabilitation react appropriately to differing labour market conditions, for example in the context of work-related treatments during rehabilitation (Bethge et al. 2018) or the transition to aftercare.

We operationalized the outcome using the number of days in employment with social insurance contributions in the first calendar year. The dataset provides the number of days only by calendar year and not for other time periods. The outcome by calendar year leads to a large gap between rehabilitation and RTW measures for some individuals. This is random, and the dataset is large, so we see no bias in regression modelling. The social insurance contributions from employment in particular are one advantage of the database, because they are reported by employers and are therefore without bias. The RTW can be defined in many ways (Young et al. 2005; Nübling et al. 2016). We have discussed and empirically considered alternative RTW operationalizations elsewhere (Leinberger et al. 2023). Since these are highly correlated, labour market correlations are likely to be largely independent of the operationalization choice of RTW. We demonstrated this in additional analyses for a dichotomous outcome and for days in employment in the second calendar year. The results may be generalized to other outcomes, as patient-reported outcomes are highly correlated with administrative RTW data (Nübling et al. 2017).

For control variables, we included socio-demographics, rehabilitation characteristics, and employment biographies, as well as an aggregated variable on the department level. The selection is based on theoretical considerations, especially the noninfluence of rehabilitation departments, as well as empirical relevance. The model quality in M3 and M4 can be rated as good. Assuming that unknown treatment quality is likely to account for a considerable part of residual confounding, the essential predictors seem to be included in the model.

Observational studies are limited. Because of the non-experimental design, self-selection into the treatment might be an issue. Possibly omitted but relevant control variables could lead to bias and limit causal interpretation.

Self-selection into the treatment is minimized, because there is a standardized path from the application to the start of rehabilitation with a legal right to access. But we have no information about subjective rehabilitation needs, refused applications and underutilization of rehabilitation.

We use the regional unemployment rate. Other labour market characteristics can be omitted in our opinion because the variance components for the labour market are empirically close to zero, indicating a very good operationalization of the labour market. Other operationalizations (including economic structure, commuting links, unemployment structure, and unemployment trend) have been tested elsewhere, but have proved to be of low importance (not displayed here). Moreover, this confirms the regional unit chosen, which was homogenized in terms of commuting links. For alternative regional units, such as political-administrative or settlement-structured regions, remodeling of commuter links might have been necessary.

Other individual factors would be of importance if they were associated with both the labour market and the outcome variable and thus altered the regression estimators. We excluded diagnoses because the diagnoses mainly affect RTW but should not be associated with the labour market. Therefore, they are not important for the unbiased estimation of labour market effects. We demonstrated this by additional analyses for subgroups.

In terms of employment biography, we used employment days in the first and second year before rehabilitation, as well as the employment status in the third month before. These predictors have proved to be the main predictors of RTW. The database offers alternative options for operationalizing employment biography: variables per calendar year (days in employment, days in receipt of unemployment benefits, earnings) and status per month (yes/no: employment, unemployment, parenthood, etc.). We did not apply all these factors because they are highly correlated (multicollinearity) or biased (in the case of earnings, there is no information about part-/full-time employment and about the household income). Even with our parsimonious operationalization, the influence of the labour market was nonexistent (0.0 days in psychosomatics) or almost nonexistent (-0.4 days in orthopaedics). We have shown in additional analyses that this is robust when we add further employment biography predictors to model 3.

Occupation, industry, workload, etc., would be relevant to determine labour market effects on RTW because they are likely to be correlated with both labour market and outcome variables. For example, certain sublabour markets (e.g., for bottleneck occupations such as nurses) might differ from the general labour market (Fedorets et al. 2019). However, these characteristics are either not present in the database or are insufficiently recorded (e.g., occupation key of the last job). Unobserved heterogeneity may therefore be present and lead to bias.

The same applies to characteristics that suggest the motivation to find a job (e.g., household income, financial obligations, employment status of the partner, domestic care situation).

Other interaction effects could be significant. However, nonlinear models such as the FLR used here already implicitly model interactions and are thus less sensitive to interactions. This also partially explains the small interaction effect in M4. Since, to our knowledge, there are no substantial further interaction hypotheses, we did not test any further interactions empirically.

Reverse causality is excluded because the direction of effect is predetermined by the data structure and the temporal sequence. This means that context effects can only affect the individual and not vice versa. Moreover, the employment days are timed after the intervention and the labour market is related to the period of the intervention. In this respect, a causal inference is permissible.

The FLR estimation method applied is rather uncommonly used. However, given the bimodal distribution of the outcome variable and its limited range between 0 and 365, it is appropriate. Alternatives have been rejected for reasons presented elsewhere, such as models with zero–one-inflation (Leinberger et al. 2023).

The database fully reflects the rehabilitation programmes of the GPI with high data quality. Since only the employment biography and no socio-demographic or rehabilitation characteristics seem to influence the estimation of the labour market effect, we consider the results transferable, at least for welfare states with Bismarckian systems such as Germany (Kolmar 2007). Social insurance schemes differ according to the relationship between contributions and benefits. Bismarckian systems provide earnings-related benefits, while Beveridgean systems offer flat payments. Moreover, the findings are relatively stable across the two major indication groups and even across subgroups by diagnosis, which is why transferring them to other indication groups seems legitimate. But rehabilitation programmes of only one year were analyzed, and period effects relevant to the labour market, such as the COVID-19 pandemic or financial crises, limit the generalizability.

## Conclusion

We conclude from the findings that the influence of the labour market on RTW is small. It is largely proxied by individual employment biographies. This finding remains plausible even if the influence of the labour market differs according to these biographies. However, if no (valid) employment biographies are available, the labour market should be included in RTW analyses. Database and methods are of high quality, but because of the nonexperimental design, omitted variables could lead to bias and limit causal interpretation.

## Supplementary Information

**Additional file 1: Table S1.** Fractional logit regression models for RTW in orthopaedics (all predictors). **Table S2.** Fractional logit regression models for RTW in psychosomatics (all predictors).

## Data Availability

The data used in the present study are available from the German Pension Insurance. But restrictions apply to the availability of these data, which were used at our request for this study. Data are however available from the authors upon reasonable request and with permission of the German Pension Insurance.

## Abbreviations

AME: Average marginal effects
GPI: German pension insurance
RSD: Rehabilitation statistics database
RTW: Return to work

## Footnotes

1 AHB is a full-day outpatient or inpatient medical rehabilitation program. It is only considered in the case of certain illnesses and immediately follows inpatient hospital treatment (2 weeks after discharge at the latest).

2 Special medical program is work-related (medical-vocational oriented rehabilitation = MBOR), behavioral (behavioral orthopaedics = VMO, behavioral rehabilitation = VOR) or other.

3 We use the R package glmmTMB for the regression modelling (Brooks et al. 2017). AME for the regional unemployment rate are reported from the single-level model using the R package margins (Leeper 2021)).

4 We use linear regression coefficients because they are similar to AME based on logit/probit models (Breen et al. (2018).

5 For orthopaedics about 8% (10.5 × 2.8 days / 365 days) and for psychosomatics about 6% (10.5 × 2.2 days / 365 days), with 10.5% points range between the regions with the lowest and highest unemployment rates and AME from M2.
