# Supplementary material for: Return to work after medical rehabilitation in Germany: influence of individual factors and regional labour market based on administrative data
Source: J Labour Mark Res. 2023 Jan 20;57(1):4. doi: 10.1186/s12651-023-00330-1 (PMC9864500; doi:10.1186/s12651-023-00330-1)
Supplement: Supplementary file 1 — Additional file 1: Table S1. Fractional logit regression models for RTW in orthopaedics (all predictors). Table S2. Fractional logit regression models for RTW in psychosomatics (all predictors). [file 12651_2023_330_MOESM1_ESM.pdf]

Supplementary information

Table S1: Fractional logit regression models for RTW in orthopaedics (all predictors)

	M1		M2		M3		M4	
	b	s.e	b	s.e	b	s.e	b	s.e
(intercept)	1.063 ***	0.024	1.920 ***	0.031	1.514 ***	0.080	1.528 ***	0.080
unemployment rate (UR)	-0.037 ***	0.004	-0.040 ***	0.005	-0.010 **	0.004	-0.013 ***	0.004
gender [female], ref. [not female]			-0.057 ***	0.009	-0.026 **	0.010	-0.025 *	0.010
age [< 25], ref. [56-60]			-0.494 ***	0.042	0.530 ***	0.050	0.531 ***	0.050
[25-30]			-0.123 ***	0.030	0.312 ***	0.034	0.312 ***	0.034
[31-35]			-0.011	0.025	0.290 ***	0.028	0.289 ***	0.028
[36-40]			0.074 ***	0.021	0.336 ***	0.024	0.336 ***	0.024
[41-45]			0.215 ***	0.018	0.405 ***	0.021	0.404 ***	0.021
[46-50]			0.331 ***	0.014	0.440 ***	0.016	0.440 ***	0.016
[51-55]			0.306 ***	0.012	0.363 ***	0.014	0.363 ***	0.014
[61-65]			-0.979 ***	0.012	-1.009 ***	0.014	-1.009 ***	0.014
marital status [single], ref. [married]			-0.118 ***	0.013	-0.055 ***	0.015	-0.055 ***	0.015
[divorced]			-0.109 ***	0.013	-0.024	0.014	-0.024	0.014
[widowed]			-0.098 ***	0.026	-0.081 **	0.028	-0.079 **	0.028
[n.a.]			-0.161 ***	0.037	0.038	0.042	0.037	0.042
country of birth*nationality [other*other], ref. [German*German]			-0.464 ***	0.022	-0.284 ***	0.025	-0.283 ***	0.025
[other*German]			-0.125 ***	0.017	-0.152 ***	0.019	-0.152 ***	0.019
[German*other]			-0.146 ***	0.027	-0.054	0.030	-0.054	0.030
vocational education [no/n.a.], ref. [yes]			-0.521 ***	0.009	-0.275 ***	0.010	-0.276 ***	0.010
German states [new states], ref. [former West]			0.023	0.026	0.014	0.021	0.014	0.021
post-hospital curative treatment [yes], ref. [no]			-0.233 ***	0.010	-0.311 ***	0.011	-0.311 ***	0.011
special medical programmes [work-related], ref. [normal]			-0.681 ***	0.018	-0.345 ***	0.018	-0.345 ***	0.018
[other]			-0.060	0.051	-0.058	0.054	-0.058	0.054
[behavioural]			-0.253 ***	0.037	-0.175 ***	0.032	-0.175 ***	0.032
additional payment claim [no], ref. [yes]			-0.344 ***	0.012	-0.003	0.012	-0.003	0.012
application for reduced earning capacity pension [yes], ref. [no]			-2.233 ***	0.031	-1.579 ***	0.034	-1.580 ***	0.034
number of prior rehabs 4 years before [0], ref [≥3]			-0.349 ***	0.012	0.009	0.014	0.010	0.014
[1]			-0.399 ***	0.015	0.042 *	0.018	0.042 *	0.018
[2]			-0.731 ***	0.028	0.170 ***	0.032	0.172 ***	0.032
employment days one year before [<50], ref. [≥350]					-1.563 ***	0.023	-1.564 ***	0.023
[50-99]					-1.117 ***	0.035	-1.117 ***	0.035
[100-149]					-0.941 ***	0.032	-0.942 ***	0.032
[150-199]					-0.836 ***	0.027	-0.837 ***	0.027
[200-249]					-0.726 ***	0.023	-0.726 ***	0.023
[250-299]					-0.562 ***	0.020	-0.562 ***	0.020
[300-349]					-0.465 ***	0.016	-0.466 ***	0.016
employment days two years before [<50], ref. [≥350]					-1.018 ***	0.022	-1.018 ***	0.022
[50-99]					-0.834 ***	0.041	-0.833 ***	0.041
[100-149]					-0.768 ***	0.039	-0.768 ***	0.039
[150-199]					-0.688 ***	0.031	-0.688 ***	0.031
[200-249]					-0.605 ***	0.028	-0.606 ***	0.028
[250-299]					-0.443 ***	0.023	-0.443 ***	0.023
[300-349]					-0.366 ***	0.021	-0.365 ***	0.021
prerehabilitation employment status (employed) on department level					0.009 ***	0.001	0.009 ***	0.001
prerehabilitation employment status [not employed], ref. [employed]					-1.272 ***	0.012	-1.338 ***	0.029
UR * prerehabilitation employment status [not employed]							0.011 *	0.004

random effects				
$\tau_{00, \text{labour market region}}$	0.01	0.01	0.00	0.00
$\tau_{00, \text{rehabilitation departments}}$	0.05	0.05	0.01	0.01
Pseudo-R^2	0.019	0.144	0.363	0.363
AIC	369,386	339,216	282,902	282,898

M = model, b = coefficients, s.e = standard error, ref. = reference, τ_{00} = variance component of labour market region or rehabilitation department, R^2 = square of the correlation between the model's predicted values and the actual values, AIC = Akaike-criterion; method is cross-classified fractional logit regression with $n_{\text{labour market region}}$ = 257, $n_{\text{rehabilitation departments}}$ = 589, n_{patients} =305,980,
* $p<0.05$, ** $p<0.01$, *** $p<0.001$.

Table S2: Fractional logit regression models for RTW in psychosomatics (all predictors)

	M1		M2		M3		M4	
	b	s.e	b	s.e	b	s.e	b	s.e
(intercept)	0.383 ***	0.039	1.234 ***	0.042	1.229 ***	0.092	1.280 ***	0.093
unemployment rate (UR)	-0.019 ***	0.004	-0.025 ***	0.005	-0.002	0.004	-0.011 *	0.005
gender [female], ref. [not female]			0.038 **	0.014	0.007	0.016	0.007	0.016
age [< 25], ref. [56-60]			-0.724 ***	0.062	0.290 ***	0.071	0.291 ***	0.071
[25-30]			-0.253 ***	0.041	0.200 ***	0.046	0.201 ***	0.046
[31-35]			-0.171 ***	0.034	0.244 ***	0.038	0.244 ***	0.038
[36-40]			-0.039	0.029	0.289 ***	0.032	0.289 ***	0.032
[41-45]			0.105 ***	0.026	0.354 ***	0.028	0.354 ***	0.028
[46-50]			0.219 ***	0.021	0.339 ***	0.024	0.339 ***	0.024
[51-55]			0.214 ***	0.019	0.286 ***	0.022	0.286 ***	0.022
[61-65]			-0.860 ***	0.024	-0.852 ***	0.026	-0.851 ***	0.026
marital status [single], ref. [married]			-0.149 ***	0.019	-0.075 ***	0.021	-0.074 ***	0.021
[divorced]			-0.086 ***	0.018	-0.015	0.020	-0.014	0.020
[widowed]			0.079 *	0.037	0.091 *	0.041	0.090 *	0.041
[n.a.]			-0.338 ***	0.045	0.056	0.051	0.057	0.051
country of birth*nationality [other*other], ref. [Germany*German]			-0.553 ***	0.038	-0.456 ***	0.042	-0.456 ***	0.042
[other*German]			-0.250 ***	0.029	-0.257 ***	0.032	-0.258 ***	0.032
[Germany*other]			-0.153 ***	0.042	-0.116 *	0.046	-0.115 *	0.046
vocational education [no/n.a.], ref. [yes]			-0.554 ***	0.015	-0.338 ***	0.016	-0.338 ***	0.016
German states [new states], ref. [former West]			0.065 *	0.028	0.137 ***	0.027	0.136 ***	0.027
post-hospital curative treatment [yes], ref. [no]			-0.301	0.286	-0.703 *	0.309	-0.708 *	0.309
special medical programmes [work-related], ref. [normal]			-0.930 ***	0.018	-0.482 ***	0.020	-0.482 ***	0.020
[other]			-0.266 **	0.098	-0.177	0.106	-0.177	0.106
[behavioural]			-1.138 ***	0.320	-1.014 **	0.352	-1.015 **	0.352
additional payment claim [no], ref. [yes]			-0.294 ***	0.020	-0.059 *	0.023	-0.060 **	0.023
application for reduced earning capacity pension [yes], ref. [no]			-2.436 ***	0.032	-1.741 ***	0.034	-1.741 ***	0.034
number of prior rehabilitation interventions 4 years before [0], ref [≥3]			-0.345 ***	0.020	-0.079 ***	0.022	-0.078 ***	0.022
[1]			-0.437 ***	0.031	-0.101 **	0.034	-0.099 **	0.034
[2]			-0.552 ***	0.068	-0.044	0.077	-0.042	0.077
employment days one year before [<50], ref. [≥350]					-1.081 ***	0.031	-1.082 ***	0.031
[50-99]					-0.944 ***	0.043	-0.943 ***	0.043
[100-149]					-0.826 ***	0.040	-0.826 ***	0.040
[150-199]					-0.664 ***	0.034	-0.664 ***	0.034
[200-249]					-0.582 ***	0.031	-0.581 ***	0.031
[250-299]					-0.543 ***	0.029	-0.543 ***	0.029
[300-349]					-0.479 ***	0.025	-0.478 ***	0.025
employment days two years before [<50], ref. [≥350]					-0.959 ***	0.034	-0.959 ***	0.034
[50-99]					-0.794 ***	0.057	-0.795 ***	0.057
[100-149]					-0.528 ***	0.053	-0.527 ***	0.053
[150-199]					-0.600 ***	0.045	-0.600 ***	0.045
[200-249]					-0.495 ***	0.041	-0.495 ***	0.041
[250-299]					-0.436 ***	0.036	-0.436 ***	0.036
[300-349]					-0.327 ***	0.031	-0.327 ***	0.031
prerehabilitation employment status (employed) on department level					0.010 ***	0.002	0.010 ***	0.002
prerehabilitation employment status [not employed], ref. [employed]					-1.229 ***	0.018	-1.341 ***	0.042
UR * prerehabilitation employment status [not employed]							0.018 **	0.006

random effects				
$\tau_{00, \text{labour market region}}$	0.01	0.01	0.00	0.00
$\tau_{00, \text{rehabilitation departments}}$	0.13	0.12	0.04	0.04
Pseudo-R^2	0.029	0.206	0.386	0.386
AIC	157,162	138,530	117,661	117,655

M = model, b = coefficients, s.e = standard error, ref. = reference, τ_{00} = variance component of labour market region or rehabilitation department, R^2 = square of the correlation between the model's predicted values and the actual values, AIC = Akaike-criterion; method is cross-classified fractional logit regression with $n_{\text{labour market region}}$ = 257, $n_{\text{rehabilitation departments}}$ = 202, n_{patients} =117,386,
* $p<0.05$, ** $p<0.01$, *** $p<0.001$.
